# From mutations to mechanisms and dysfunction via computation and mining of protein energy landscapes

**DOI:** 10.1186/s12864-018-5024-z

**Published:** 2018-09-24

**Authors:** Wanli Qiao, Nasrin Akhter, Xiaowen Fang, Tatiana Maximova, Erion Plaku, Amarda Shehu

**Affiliations:** 1 Department of Statistics, George Mason University, Fairfax, 22030 VA USA; 2 Department of Computer Science, George Mason University, Fairfax, 22030 VA USA; 3 Department of Electrical Engineering and Computer Science, The Catholic University of America, Washington D.C., 20064 USA; 4 Department of Bioengineering, George Mason University, Fairfax, 22030 VA USA; 5 School of Systems Biology, George Mason University, Manassas, 20110 VA USA

**Keywords:** Protein dysfunction, Pathogenic mutations, Equilibrium dynamics, Energy landscape, Landscape reconstruction, Basins, Saddles, Landscape mining

## Abstract

**Background:**

The protein energy landscape underscores the inherent nature of proteins as dynamic molecules interconverting between structures with varying energies. Reconstructing a protein’s energy landscape holds the key to characterizing a protein’s equilibrium conformational dynamics and its relationship to function. Many pathogenic mutations in protein sequences alter the equilibrium dynamics that regulates molecular interactions and thus protein function. In principle, reconstructing energy landscapes of a protein’s healthy and diseased variants is a central step to understanding how mutations impact dynamics, biological mechanisms, and function.

**Results:**

Recent computational advances are yielding detailed, sample-based representations of protein energy landscapes. In this paper, we propose and describe two novel methods that leverage computed, sample-based representations of landscapes to reconstruct them and extract from them informative local structures that reveal the underlying organization of an energy landscape. Such structures constitute landscape *features* that, as we demonstrate here, can be utilized to detect alterations of landscapes upon mutation.

**Conclusions:**

The proposed methods detect altered protein energy landscape features in response to sequence mutations. By doing so, the methods allow formulating hypotheses on the impact of mutations on specific biological activities of a protein. This work demonstrates that the availability of energy landscapes of healthy and diseased variants of a protein opens up new avenues to harness the quantitative information embedded in landscapes to summarize mechanisms via which mutations alter protein dynamics to percolate to dysfunction.

## Background

Proteins are ubiquitous biological macromolecules (biomolecules) found in nearly all processes that maintain and replicate a living cell. These biomolecules are inherently dynamic, switching/interconverting between shapes/conformations with different potential energies. These interconversions regulate interactions of a protein with different molecular partners and by doing so regulate allosteric signaling, catalysis, and other cellular processes that occur on 0.1−10Å length scales and nanosecond-seconds time scales [1].

A protein energy landscape organizes the conformations accessible by a protein by their potential energies, revealing local landscape structures, such as basins and barriers. Basins correspond to thermodynamically-stable and semi-stable conformational (macro-)states; barriers are local landscape structures that separate basins and thus control basin-to-basin diffusions that correspond to state-to-state interconversions [2, 3]. Together, these local structures control the dynamics of a protein and are key to a protein’s ability to regulate its molecular interactions and so its function in the cell [1, 4].

Many human disorders (including cancer) are proteinopathies driven by DNA mutations that percolate to protein dysfunction by affecting the state-to-state interconversions via which a protein regulates interactions with molecular partners [5]. Such mutations change the energy landscape and, in particular, the local landscape structures that control the equilibrium dynamics [2]. Thus, it is highly desirable to quantify changes to the energy landscape in response to a mutation. This task is generally infeasible [6].

In principle, the protein energy landscape contains the information needed to characterize and relate protein equilibrium dynamics to function [7, 8]. However, state-to-state interconversions may span spatial and temporal scales of several orders of magnitude [6]. Due to the disparate scales involved, no wet- nor dry-laboratory techniques can fully and on their own reconstruct energy landscapes of any protein of interest [6, 9].

The challenges with reconstructing protein energy landscapes are twofold. First, due to the high dimensionality of the protein conformation space (space of all possible conformations), only randomized search algorithmic frameworks are viable [10]. Such frameworks probe the landscape one sample (conformation-energy pair) at a time, and thus obtain a discrete, sample-based representation of a landscape. Whether based on the Molecular Dynamics (MD) or Monte Carlo (MC) template, these frameworks operate under the umbrella of global optimization and are thus prone to premature convergence, which is not desirable when the objective is to obtain a broad view of the landscape so as not to miss regions of importance for the dynamics of the protein under investigation. The interested reader can learn more about these frameworks and their challenges with obtaining detailed, sample-based representations of protein energy landscapes in a recent review in [6]. The second challenge with reconstructing a protein energy landscape is due to the utilization of a discrete, sample-based representation; that is, given a set of samples spread over an invisible landscape, the challenge is to uncover the underlying organization of the samples in terms of the unknown basins and basin-separating barriers.

The contribution of this paper is in addressing the challenge of reconstructing an energy landscape by utilizing a discrete, sample-based representation for it. The ability to do so relies on recent methodological advances that are making it possible to obtain detailed, sample-based representations of energy landscapes for proteins with sufficient, wet-laboratory, conformational data (the “Related work” section summarizes these advances). Specifically, these methods are able to feasibly compute detailed, sample-based representations of energy landscapes for different variants of a protein of interest [11–14].

We present two novel methods that analyze a given set of energy-evaluated samples and automatically identify the basins containing the samples and the basin-separating saddles. The methods employ concepts from topological and statistical analysis of spatial data. The first method operationalizes a basin-driven approach, first identifying basins and then utilizing working definitions of saddle points to indicate basin-separating saddles. The second method utilizes a saddle-driven approach, first identifying saddles via precise mathematical formulations and then detecting the basins separated by them. Both methods expose the deepest point in a basin, which, alongside with identified saddles, allow extracting quantitative features/descriptors of a landscape. The latter, as we demonstrate here, permit quantitative comparison of landscapes of healthy and pathogenic variants of a protein and open up interesting avenues into landscape mining techniques and formulation of thermodynamics-based hypotheses on how mutations percolate to (dys)function.

The next section places the proposed landscape analysis methods in the context of related work. The methods are detailed in the “Methods” section. The “Results” section evaluates the proposed methods on many sequence variants of an enzyme central to human biology. The evaluation exposes several mechanisms of interest by which oncogenic and non-oncogenic but syndrome-causing mutations alter features of the energy landscape and in turn impact the healthy/wildtype (WT) equilibrium dynamics. An interesting avenue is also related on how the proposed methods open the way for machine learning approaches to operate over energy landscapes. Drawn landscape descriptors are correlated with wet-laboratory measurements, furthering biological knowledge. The “Discussion” section discusses the implications of our findings, and the “Conclusion” section concludes this paper with a summary of the contributions and their relevance for understanding protein function.

### Related work

We first summarize recent methodological advances that allow to obtain detailed, sample-based representations of protein energy landscapes and so allow the methods proposed in this paper to reconstruct energy landscapes from such discrete representations. We then provide preliminaries on local landscape structures and summarize pertinent related work in detecting such structures.

#### Sample-based representations of protein energy landscapes

Recent methodological advances are allowing the reconstruction of energy landscapes of medium-size proteins in the presence of wet-laboratory-resolved conformations [14]. The issue of disparate spatio-temporal scales is circumvented by exploiting the information available in wet-laboratory-resolved conformations of healthy and diseased (pathogenic) forms/variants of a protein. This is viable for many proteins of central importance to human biology, as steady attention from wet laboratories on such proteins has resulted in many conformations deposited for them in the Protein Data Bank (PDB) [15].

In particular, since 2015, various stochastic optimization algorithms have been proposed and refined that delay premature convergence and so obtain detailed, sample-based representations of energy landscapes of various medium-size proteins with reasonable computational budgets) [11–14, 16–18]. Sampling of conformations takes place over a carefully-selected variable space. The variables are extracted via Principal Component Analysis (PCA) of experimentally-known conformations collected from the PDB for a protein’s healthy and diseased variants. The latest molecular mechanics-based energy functions in the Amber suite are integrated [12].

The ability of these algorithms to compute detailed, sample-based representations of Amber energy landscapes feasibly (a few CPU days) for different variants of a protein of interest [12] allows the investigation in this paper on reconstructing, summarizing, and comparing landscapes to reveal the impact of mutations on dysfunction. Specifically, the data we utilize here are obtained with the SoPriMp algorithm [17]. The SoPriM algorithm [17] and its faster version, SoPriMp [11] do not directly operate in the conformation space of a protein, so as to circumvent the dimensionality issue, but instead compute samples that represent conformations in a low-dimensional variable space of principal components (PCs). Fast transformations between the variable space and the all-atom conformation space allow the algorithms to obtain low-energy, all-atom conformations of a protein sequence of interest. The interested reader can learn more about these algorithms and their evaluation in Ref. [11]. In this paper, the samples analyzed by the proposed landscape analysis methods are generated by SoPriMp (due to its higher exploration capability [17]) and correspond to computed, all-atom conformations evaluated with the Amber ff14SB forcefield.

#### Local landscape structures

Suppose *d* (*d*≥1) variables are selected to describe a protein conformation. Then the range of possible values of the *d* variables is the underlying domain *D* of the energy landscape, which is a subset of $\mathbb {R}^{d}$. In a more general sense, a landscape can be understood as a function defined on a multivariate domain. Hence, landscape analysis is related to many other disciplines, where spatial data are available. For example, the topology summarization of cosmological data is important in understanding the evolution of our universe [19].

Specifically, for an energy function *e* which maps *D* to $\mathbb {R}$, let *e*_min_= min*x*∈*De*(*x*) and *e*_max_= max*x*∈*De*(*x*). For any energy level *c* between *e*_min_ and *e*_max_, the set *L*(*c*)={*x*∈*D*:*e*(*x*)≤*c*} is called a sublevel set (or level set for brevity) of *e* and represents the subregion of *D* with energy at or below *c*. Level sets have been extensively studied in the mathematics and statistics literature. In particular, the concept of level sets plays a critical role in the recent advancement of topological data analysis (TDA) through persistent homology [20, 21]. In fact, our level set-based approach is similar in spirit to TDA, but our focus is on the geometry rather than the topology of energy landscapes.

Specifically, we are interested in how the shape of *L*(*c*) evolves as the level *c* changes, as this dependence reflects unique characteristics of a landscape. If for some energy level *c*, a connected component of *L*(*c*) has a saddle point on its boundary, then this connected component is called a basin. By this definition, a basin is a low-energy region containing at least one local minimum of the energy *e*. The various basins of the same energy landscape are organized in a hierarchical global structure. For example, two adjacent basins can be connected via a common saddle point; a smaller basin can be nested in a larger one. It is desirable for an energy landscape analysis method to automatically discover and organize such a global structure.

Due to the connection between basins and the geometric concepts including level sets and saddle points, in this paper we operationalize two different approaches in two novel methods to organizing the hierarchical structure of basins, one based on level sets (to which we refer as *basin-driven*), and the other based on saddle points (to which we refer as *saddle-driven*). We note that basins are also related to some other geometric features, such as ridges [22, 23], and they are also the focus of complementary approaches. For instance, a recent method analyzes a nearest-neighbor graph embedding of protein conformations, seeking critical points in the graph that are then utilized as working definitions of saddles and local minima onto which neighboring points are mapped to constitute basins [24]. The basin-driven method we propose here analyzes the smoothed energy landscape. This allows addressing both the ruggedness of the landscape and the non-uniform sampling density obtained from MD-, MC-, or other more powerful sampling-based methods that yield discrete, sample-based representations of landscapes. In addition, the basins identified in this work are subregions of the landscape itself with clear boundaries that allow both visualization and extraction of quantitative descriptors for feature-based summarizations of landscapes.

Searching for saddle points has also been important for spatial data analysis. In particular, the reduced gradient curve following (RGF) approach originated in a computational chemistry setting to extract saddle points [25, 26]. In this paper, we leverage mathematical formulations and techniques from the RGF approach to find *all* the saddle points on a protein energy landscape, which we then additionally link to basins.

## Methods

From now on, we refer to the two novel methods described below as Basin-Driven_Reconstruction (BDR) and Saddle-driven_Reconstruction (SDR). Both methods explicitly reconstruct a landscape by finding basins and saddles. BDR first finds basins, using precise mathematical formulations derived from the level set approach summarized above, and then uses working definitions of saddle points to detect saddles. SDR first finds saddles, using precise mathematical formulations derived from the reduced-gradient curve approach, and then uses identified saddles (and local minima) to detect basins.

The application of both methods here is limited to analysis of three-dimensional (3D) samples, using as observations the first two coordinates (PC1, PC2) of SoPriMp-computed samples and the Amber ff14SB energy values of the structures corresponding to the samples. In principle, one can utilize all coordinates (typical numbers range from 10 to 25 PCs), but the dimensionality adds to the computational cost of the analysis. It is worth noting that in cases where SoPriMp can be employed to obtain sample-based representations of energy landscapes, the top two PCs capture more than 50% of the conformation variance, and the top three capture more than 70% of the variance [11–14, 17, 18]. The emphasis on reasonable computational costs is due to the objective to apply BDR or SDR in comparative analysis settings that screen numerous variants of a protein in search of landscape features to learn the impact of mutations on dysfunction.

### Boundaries and landscape smoothing

**Fig. 1:**
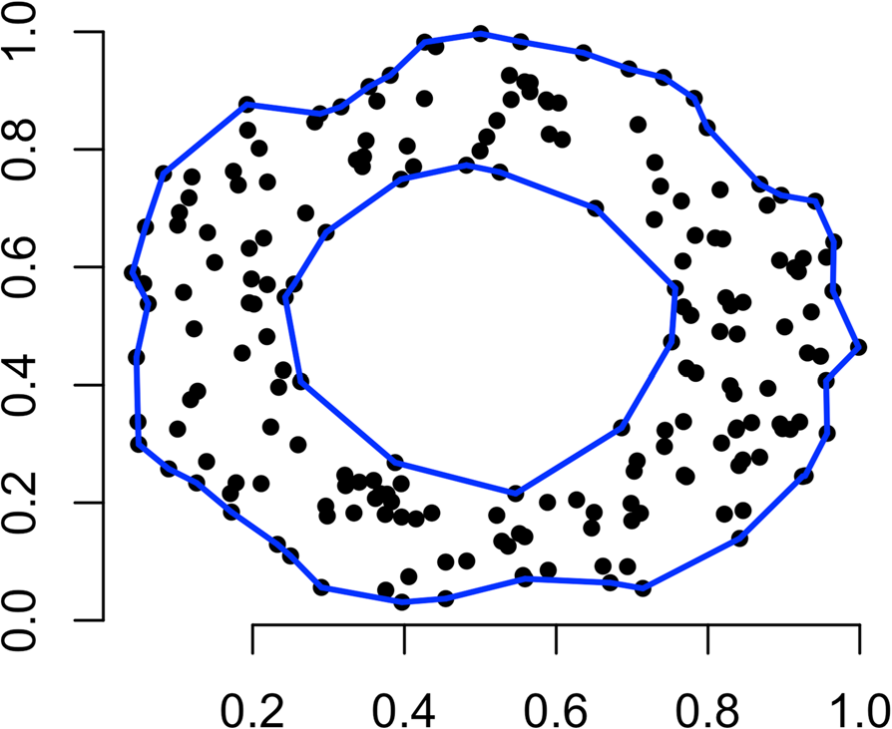
Both proposed methods first find the alpha-convex shape that encapsulates samples, illustrated here on randomly-generated 2D points

Both BDR and SDR preprocess the samples as follows. First, the *α*-convex shape [27, 28], a generalization of the convex hull, is computed on the PC1-PC2 sample locations. This is illustrated in Fig. 1. Then, a 2D grid is then defined over the samples that fall in the alpha-convex shape. A parameter *δ*_1_ is used to control the distance between adjacent grid points. The resulting collection of grid points is denoted as *S*_*max*_.

**Fig. 2:**
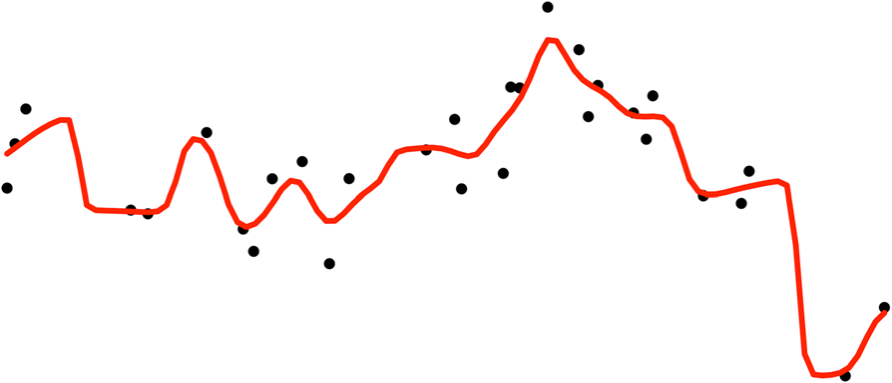
Once a grid is imposed on points in the computed alpha-convex shape, the energies of the grid points are estimated via kernel regression. This is illustrated here on 1d points

Kernel regression is used to estimate the energy of each grid point. Kernel regression is a central smoothing technique in spatial data analysis, as illustrated in Fig. 2. Protein energy landscapes reconstructed with all-atom energy functions, such as Amber ff14SB here (and others), are known to be overly rugged [6]. Kernel regression is a mechanism to reduce the ruggedness and address the non-uniform density of samples.

As reported in the “Results” section, we evaluate different kernels on the trade-off between accuracy and computational speed. Three kernels are available in python’s sklearn library, Gaussian, Epanechnikov, and Tricube; they are compared to one another and our own implementation of a bounded-support Gaussian kernel. In each case, the energy estimate of each grid point *x* is the weighted average of energies of observed samples (PC1-PC2 locations). Specifically, the sklearn kernels sum up the contribution from each sample in the computed alpha-convex shape and weight the contribution of each sample based on the sample’s distance (in PC1-PC2 space) to the grid point whose energy is being estimated. Our own implementation of a bounded support kernel considers only samples within an *h*-radius neighborhood centered at a grid point (note that the amount of smoothing is controlled by *h*); the kernel utilizes a kd-tree, a proximity-query data structure to expediently find the nearest neighbors (among samples) of a grid point, thus yielding computational savings.

Once the energies of grid points have been estimated, each of the proposed BDR and SDR methods proceeds differently. We now relate details.

### The BDR method: basin-driven landscape reconstruction

Let the minimum and maximum energy over the (grid) points in the grid *S*_*max*_ be *c*_*max*_ and *c*_*min*_. Starting from *c*=*c*_*max*_, BDR iteratively decreases the energy level *c* by a small step *δ*_2_, detecting splitting of basins and storing resulting basins and saddles in a list *Ω*; the latter is utilized for further visualization and quantitative analysis and comparison of landscapes.

Algorithm 1 shows pseudocode for a recursive implementation of the BDR method. The initial arguments to BDR are all the grid points *S*=*S*_*max*_, the maximum energy *c*=*c*_*max*_, and an empty *Ω* list of yet-to-be-found basins and saddles.


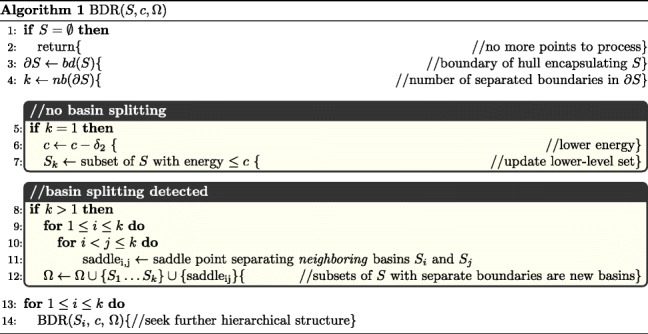


Lines 3–4 in Algorithm 1 show that on a given collection *S* of grid points (*S* is initialized to *S*_*max*_), BDR calculates the *k* boundaries of *S* (using the *α*-convex hull). If *k*=1 (no basin splitting has been detected), BDR decreases *c* by *δ*_2_ and focuses on the corresponding level set (lines 5–7); the grid locations that meet the energetic threshold are updated (line 7). Otherwise, if *k*>1, basin splitting has been detected (lines 8–12); the new *S*_1_…*S*_*k*_ components with separate boundaries are basins that are added to *Ω*. Saddles *saddle*_*i*,*j*_ are defined for each pair of neighboring basins *S*_*i*_ and *S*_*j*_. The minimum distance between vertices on polygonal boundaries of different basins *S*_*i*_,*S*_*j*_ is computed; if this distance is not above a threshold *d*_*th*, *S*_*i*_ and *S*_*j*_ are deemed neighboring basins, and the middle point of the minimum-length line is estimated to be the saddle point *saddle*_*ij*_. The identified saddles are also added to *Ω*. Whether *k*=1 or *k*>1, BDR proceeds to identify further, possible hierarchical structure in the landscape (lines 13–14). The method terminates when no more grid locations are left (lines 1–2).

In the interest of clarity, the pseudocode in Algorithm 1 does not show modifications made to lower the computational cost. One modification concerns avoiding computing the dense boundaries in the high-energy regions, which is both time-consuming and irrelevant, since the basins in which we are most interested are in the low-energy regions of the landscape (that is where we want most detail). This is achieved by making use of a cutoff point *c*_0_. If *c*≤*c*_*min*_+*c*_0_, no adjustment of BDR occurs. Else, a threshold *n*_0_ is used; if the number of grid points within any basin *S*_*i*_ is less than *n*_0_, BDR does not dig further into *S*_*i*_.

The value of *δ*_2_ in BDR affects the overall computational cost, as well. There is a trade-off between keeping the computational budget reasonable and capturing the exact moment when a basin splits. Rather than always lowering the energy level by *δ*_2_ (line 6 in Algorithm 1), a jump test is performed first by decreasing *c*by *mδ*_2_ for some moderately large *m*. If the resulting lower level set at *c*−*mδ*_2_ splits into smaller basins, then the exact moment of basins splitting is missed, and BDR reverts back to *c*−*δ*_2_. Otherwise, no interesting events occur between *c* and *c*−*mδ*_2_; so, BDR directly moves down to the level *c*−*mδ*_2_ and continues with the jump test until the test fails. We note that jumps are only applied for *c*>*c*_*min*_+*c*_0_, as computational costs decrease with *c*≤*c*_*min*_+*c*_0_ (computation time correlates negatively with *c*). It is possible that some very small and shallow basins can quickly appear and disappear in a jump between *c* and *c*−*mδ*_2_. In this sense, the parameter *m* has a similar effect to *n*_0_ in terms of ignoring small basins. These modifications to BDR recognize that it is best to focus computational resources (and so keep all details) in the region with energy level *c*≤*c*_*min*_+*c*_0_.

### The SDR method: saddle-driven landscape reconstruction

The foundation of the SDR method is the fact that adjacent basins are connected at saddle points. The goal of searching for the splitting moments in BDR can also be achieved by direct detection of saddle points. Once saddle points are detected, the corresponding basins can be found by tracking the boundaries of basins.

The proposed SDR method builds over the RGF approach originally presented in [25, 26] to compute saddle points. The RGF approach utilizes the knowledge that all critical points of a smooth function have to have zero first-order derivatives (gradient in a multivariate setting), and the critical points can be viewed as solutions to *d* equations with *d* parameters, where *d* is the dimension of the landscape. In a multi-dimensional space, if one of the equations is neglected, the solutions becomes curves that connect all the critical points. The RGF approach takes a local minimum as input, then follows the trajectory of the solution curves of the reduced-gradient equations and stops when the definition of a saddle point is satisfied (that is, the gradient evaluated at that point is close to zero, and the eigenvalues of the Hessian matrix evaluated at that point have opposite signs).

In the proposed SDR method, our goal is to find *all* the saddle points on the energy landscape so as to detect the organization of the landscape. The implementation of the SDR method shown in pseudocode in Algorithm 2 takes a grid *S* (initialized to *S*_*max*_ as for BDR). The method detects all local minima first, and then follows the reduced gradient curves starting from the identified local minima to find all saddle points.


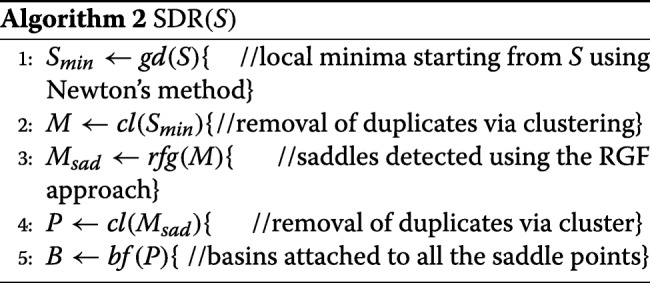


To ensure the detection of all local minima, Newton’s conjugate gradient descent method (utilized via python’s scipy library) is applied to each grid point to obtain the local minimum nearby each grid point (line 1 in Algorithm 2). It is possible that multiple estimates of a local minimum can be obtained when starting from nearby grid points. It is worth noting that in numerical programming, the notion of duplicates (which ideally request a testing for equality) is extended to include sufficiently-close points, so clustering is employed (line 2). We make use of a simple leader-clustering algorithm, where a point either forms a new cluster (becoming its representative) or is assigned to the first cluster whose representative is within *ε*; Euclidean distance is used to determine the distance between a point and a cluster representative.

The SDR method then finds all saddle points using the RGF approach on the resulting local minima (line 3). Briefly, the RGF approach proposes iterations between predictor and corrector steps to walk along a reduced gradient curve. Let a local minimum be *x*_1_; *x*_1_ is on a reduced gradient curve by definition. A tangent direction $\vec {t}$ to the reduced gradient curve can be obtained via an orthogonal vector to a column of the Hessian matrix evaluated at *x*_1_; Four options are possible in two dimensions, based on the two columns of the Hessian matrix that can be considered and the two options for the definition of an orthogonal vector. All options are followed in SDR. For a given tangent vector, the predictor step yields a point $x_{2} = x_{1} + p \cdot \vec {\hat {t}}$, where *p* is a user-defined step length, and $\vec {\hat {t}}$ is the normalized tangent vector. Since *x*_2_ deviates from the reduced gradient curve, the corrector step yields pulls it back to the reduced gradient curve by setting $x_{3} = x_{2} + \vec {c}$, where $\vec {c}$ is the correction vector orthogonal to $\vec {t}$. The exact solution for $\vec {c}$ is obtained by solving two linear equations, as detailed in the RGF approach in [26]. This iterative, prediction-correction process is now repeated from *x*_3_ and so on. The process stops when the reduced gradient curve being followed exits the grid. Points in a reduced gradient curve that are saddle points are identified via the saddle point test summarized above. Note that in numerical programming, testing for equality (e.g., in determining whether the gradient is 0) needs to make use of a very small tolerance parameter, which we denote by *ε*_*g*_.

The separation between the predictor and corrector steps can be avoided by linearly combining the two steps into one [26]. That is, *x*_3_ can be obtained directly as $x_{3} = x_{1} + \vec {d}$, where $\vec {d}$ is a vector that satisfies two linear equations outlined in [26]. In our SDR implementation, both techniques are supported.

It is possible that multiple estimates of a saddle point can be obtained when starting from different local minima, as a saddle point can be crossed by multiple reduced gradient curves; the leader clustering algorithm is utilized again in this case (line 4). The groups of duplicates are well-separated, because each group contains the estimates of the same critical point, and within-group variation is controlled by the error tolerance in the optimization and the RGF approach. Therefore, clustering can consistently find the critical points. We note that the detections of local minima and saddle points are both embarrasingly parallel processes, as the computations initiated from different starting points are independent and can be run in parallel.

With all the saddle point estimates, we can now find boundaries of basins (line 5). For each saddle point, we first compute the energy associated with it. Ideally, if one tracks along the orthogonal directions to the gradient, one should obtain the trajectory of the contour lines. However, the gradient at a saddle point is zero and thus has no well-defined orthogonal directions. To address this problem, we perform a technical treatment called “jitter” by slightly deviating from the estimated saddle points. The direction of deviation is chosen to be the eigenvector of the Hessian corresponding to the smallest eigenvalue. The chosen direction points to the inner part of the basins. Then as indicated above, following the directions orthogonal to the gradient, we can track the boundary of a basin, until a closed curve is created. In this way, for each saddle point, we find two paired basins, which are attached at the saddle point. Again this process is highly parallel, as runs can be setup in parallel for the different saddle points.

### Implementation details

The values of parameters in the BDR method are: *δ*_1_=0.1, *δ*_2_=0.3, *m*=50, *c*_0_=200, *n*_0_=100, *α*=0.15 (parameter for the *α*-convex shape), and *d*_*th*=20·*α*. Different values of *h* are analyzed (see the “Results” section). BDR has been implemented in R and python. In the R implementation, the method takes 12–24 CPU hours to complete in a single-processor setting on about 50,000 samples; slightly over half of the time is used by the kernel regression. In the python implementation, the utilization of the bounded-support Gaussian kernel cuts down this time by more than two orders of magnitude (e.g., from of 7.5 h to 80 s). The employment of the bounded-support kernel, multiple processors (4 for the results reported in the “Results” section), and the jump test described above bring down the running time of the BDR method from 12–24 h to under 2 h. In the SDR method, the values of the parameters are: *p*=0.1, *ε*=0.01, and *ε*_*g*_=0.001. SDR has been implemented in python. The bounded-support kernel and multiple processors are also employed to obtain estimated energies for grid points in an embarrassingly parallel fashion. Overall, SDR takes about 1 h on about 50,000 samples.

## Results

We evaluate BDR and SDR on samples obtained for 15 variants (sequences) of the H-Ras enzyme, which is a human cell growth regulating enzyme with mutations implicated in many human disorders that include cancer [5]. Three sets of results are related.

First, the ability of BDR to reconstruct an energy landscape is related first on the H-Ras WT. The impact of the bandwidth parameter (which serves to smooth the ruggedness) is showcased by comparing the landscape reconstructed at two different bandwidth values. The local minima detected by the SDR method are also shown on the landscapes reconstructed at the two different bandwidths. The comparison at two different bandwidths is utilized to select a bandwidth value at which to relate the rest of the results. At the selected bandwidth value, SDR is shown in action, with its reduced gradient curves, local minima, and saddles. Second, once a baseline bandwidth value is selected, landscapes are reconstructed for all 15 variants of H-Ras. The landscapes are first compared visually, and insightful observations are drawn into what mechanisms the different mutations (in the variants) employ to alter the H-Ras energy landscape with implications for H-Ras dysfunction. Third, quantitative descriptors extracted off identified basins and basin-separating saddles are then correlated to wet-laboratory biochemical measurements. This allows relating alterations of landscapes to specific biological activities in which H-Ras participates in the cell.

Before we proceed relating these three sets of results, we first summarize current biological knowledge on the H-Ras equilibrium dynamics and function, as we utilize these knowledge to evaluate and interpret the reconstructed landscapes and their characteristics.

### H-Ras dynamics and function in literature

**Fig. 3:**
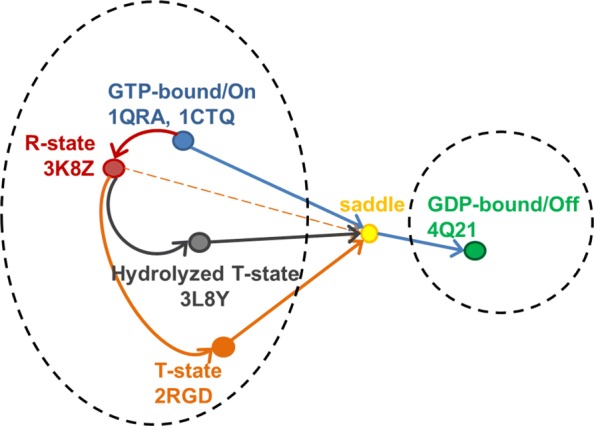
All current biological knowledge on H-Ras is summarized in this schematic, showing known and putative state-to-state interconversions

Figure 3 summarizes current knowledge on H-Ras and relates the presence of two large basins in the WT H-Ras energy landscape, one corresponding to the On or GTP-activated state and the other to the Off or GDP-activated state. The known energy barrier separating these two states is related in the schematic via a pseudo-saddle point. Prior work on obtaining a sample-based representation of the H-Ras WT energy landscape indicates that GTP-activated basin is larger, and contains many conformations reported by different wet laboratories under PDB entries 1QRA, 1CTQ, 3L8Y, 2RGD, 3K8Y, and more [12, 18]. The latter three represent known allosteric states of H-Ras, denoted as Reactive (R) and Tardy (T) in Fig. 3. Work in [29] reports interconversion between the R- and T-states and suggests these states to be more important than the On-to-Off interconversion for dysfunction in oncogenic variants of H-Ras.

### Evaluation of BDR and SDR on the reconstructed landscape of H-Ras WT

**Fig. 4:**
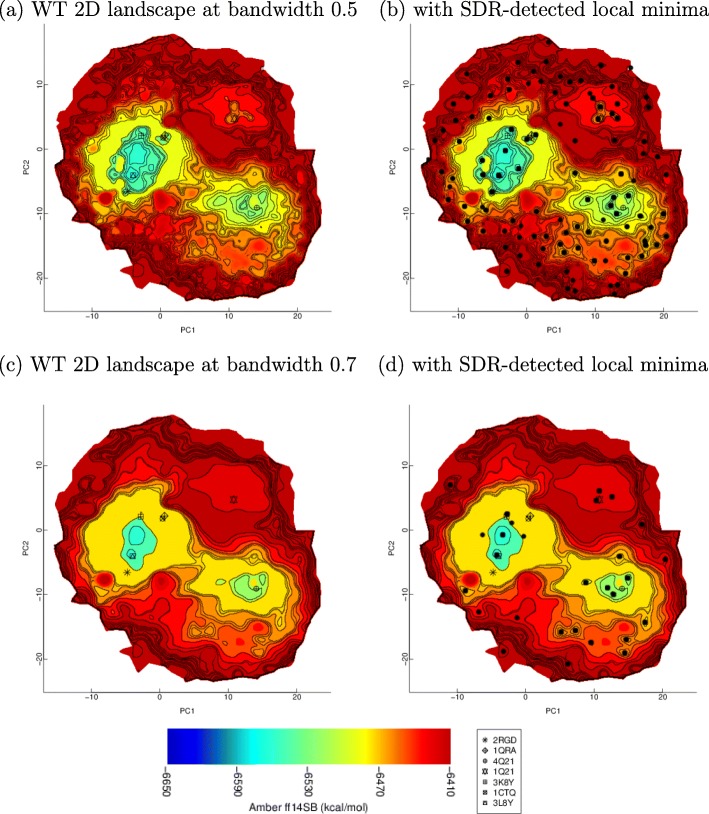
**a**-**d**: BDR-reconstructed landscape of H-Ras WT at two bandwidth values, 0.5 in **a**-**b** and 0.7 in **c**-**d**. The color coding-scheme is based on Amber ff14SB energy values estimated for every grid point as described in the “Methods” section. Symbols that annotate projections of select experimentally-known structures are also shown. Local minima detected by SDR at two bandwidth values are shown as black dots in **b** and **d**, respectively

BDR is applied to about 50,000 samples obtained via SoPriMp on the H-Ras WT sequence and used to reconstruct the H-Ras WT energy landscape at different bandwidth values (from 0.3 to 1.0). Figure 4 shows the reconstructed landscapes at bandwidth values 0.5 and 0.7. Contour lines indicate the found basins, which are color-coded via a blue-to-red color-coding scheme denoting low-to-high energies. Wet-laboratory conformations are projected onto the same space of PCs to relate detected local structures to states represented by conformations detected in the wet laboratory for different, known thermodynamically-stable and semi-stable states of H-Ras.

Juxtaposing the BDR-reconstructed H-Ras WT energy landscape at two different bandwidth values on the left panel of Fig. 4 shows the impact of the bandwidth parameter on the local structures detected on the H-Ras WT landscape. Specifically, at both bandwidth values, two large basins emerge (in light blue). Based on the co-location of projections of wet-laboratory-resolved conformations, these basins correspond to the known On and Off states, with the larger one corresponding to the On state. Moreover, at both bandwidth values, the known R- and T-states (represented by wet-laboratory-resolved conformations found under PBD entries 3K8Z, 2RGD, and 3L8Y) reside within the On basin.

The left panel of Fig. 4 shows that the higher bandwidth value allows identifying the R- and T-states as smaller basins within the GTP-activated basin, directly validating prior work that reports interconversions of these states [29]. Figure 4a-b highlights that lower bandwidth values provide more detail and expose narrow basins, such as the one populated by known GTP-activated conformations (PDB entries 1QRA and 1CTQ) within the larger On basin. The On basin is “smoothed away” at the higher bandwidth value, as seen in Fig. 4c-d. In particular, the hydrolyzed T-state (represented by the conformation under PDB entry 3L8Y) sits within the larger, GTP-activated state at the lower bandwidth value but moves just outside at the higher bandwidth value. The impact of the bandwidth is also observed on the local structures detected by SDR. The right panel of Fig. 4 shows the local minima (as black dots) on the reconstructed landscapes at bandwidth 0.5 and 0.7, respectively.

Taken together, the results suggest that bandwidth values ≥0.8 remove too many important local structures of the landscape, whereas the high level of detail obtained at bandwidth values ≤0.5 makes visualization difficult. Moreover, it is well understood that in bounded-support kernels, small support size leads to overfitting. Therefore, the rest of the results related are obtained with a bandwidth value of 0.7.

**Fig. 5:**
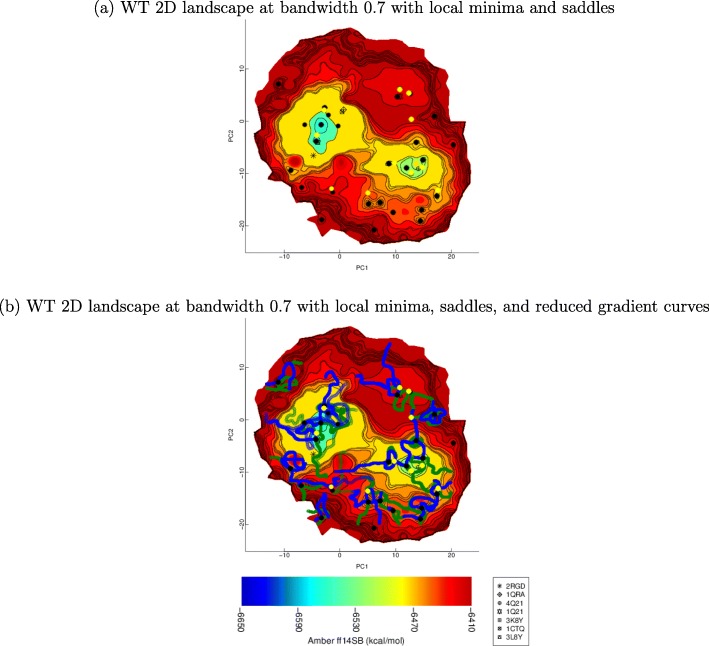
**a** SDR-obtained local minima and saddles are shown superimposed over the WT landscape at bandwidth 0.7. Local minima are drawn as black dots, and saddles are drawn as yellow dots. **b** Reduced gradient curves are additionally plotted from local minima that lead to saddles. The reduced gradient curves tracked by following the first column of the Hessian matrix are colored in green, and the curves tracked by following the second column of the Hessian matrix are colored in blue

We showcase the additional capabilities of SDR in computing saddles and reduced gradient curves over BDR. Specifically, Fig. 5a shows the local minima and the saddles found by SDR at bandwidth 0.7. Figure 5b additionally plots the reduced gradient curves that SDR tracks to find saddles from local minima, effectively showing SDR in action. We note that not all local minima lead to saddles in implementation (as can be observed in Fig. 5 for two local minima). Local minima with no close neighbors among samples are effectively in under- or poorly-sampled regions of the conformation space. Rather than expand the neighborhood radius on a per-point basis, we elect not to advance computations from such points.

We now relate results on visual and quantitative comparison of landscapes reconstructed with a bandwidth value of 0.7 to draw insight into how mutations alter landscape features and via those function in pathogenic variants of a protein.

### Visual comparison of reconstructed landscapes of variants

**Fig. 6:**
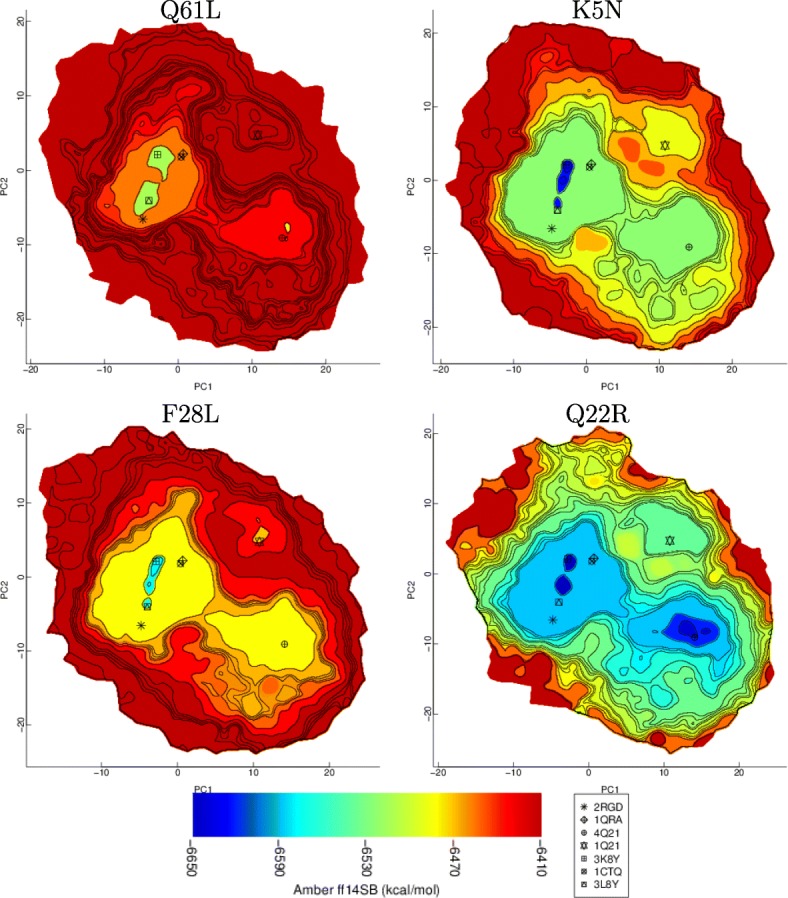
Landscapes of oncogenic (left) and syndrome-causing (right) variants (right) are shown. The color-coding scheme and the symbols annotating projections of select known structures are as in Fig. 4

Important insights can be obtained via visual comparison of reconstructed landscapes. Fifteen sets of data (each set consisting of about 50,000 samples computed by SoPriMp on a different H-Ras variant/sequence) are subjected to BDR to obtain corresponding reconstructed landscapes. Visual comparison of landscapes shows that mutations impact the size of the main (On and Off) basins, the appearance or disappearance of smaller basins within larger basins, the heights of barriers separating basins, and/or the split/separation of basins. Figure 6 relates representative results by showing reconstructed landscapes of representative oncogenic (on the left panel) and non-oncogenic but syndrome-causing (on the right panel) variants of H-Ras. These landscapes are visually compared to the WT landscape (shown in Fig. 4).

The comparison (including more landscapes not shown here) allows making the following summary observations. The barrier separating the On and Off basins is higher in the oncogenic variants than in the WT; the landscape of the Q61L variant (where Q at position 61 in the H-RAs WT has been replaced with L in this oncogenic variant) is a representative of this mechanism employed by oncogenic mutations. Moreover, in oncogenic variants the Off basin shrinks or disappears entirely (Q61L and F28L illustrate this mechanism). In addition, the On basin splits, separating the R- and T-states (see Q61L).

The elevation of the barrier between the On and Off states or the disappearance or shrinkage of the Off basin rigidify H-Ras (making interconversions between the On and Off states energetically costly and so slowing the diffusion between the two states). This rigidification has been reported and is so validated by prior work [13]. Comparison of the reconstructed landscapes exposes a novel feature that has not been captured by prior work, namely, the separation of the R- and T-states in the oncogenic variants of H-Ras (due to basin splitting). Prior wet-laboratory work suggests that the R-to-T interconversion in H-Ras is central to function. The separation of the R- and T-states observed in the oncogenic landscapes indeed provides further evidence of an interesting mechanism via which oncogenic mutations percolate to dysfunction, namely, by disrupting the allosteric switch.

The right panel of Fig. 6 shows representative reconstructed landscapes of syndrome-causing H-Ras variants. Degeneracy is observed: the Off basin leaks into other regions, merges with the On basin, or spills over the landscape (Q22R illustrates this degeneracy). In summary, on the syndrome-causing variants, more regions become energetically-favorable, including a smaller third basin (see Q22R) that corresponds to a semi-stable state off the pathway that connects the On and Off basins. The degeneracy observed in syndrome-causing variants suggests an interesting complementary mechanism for dysfunction via delay of the On-to-Off diffusion (by diffusions within the Off basin).

### Quantitative comparison and mining of reconstructed landscapes of variants

The reconstructed landscapes can be summarized via quantitative descriptors as follows. An experimentally-known state (On, Off, R-, etc.) can be mapped to the global minimum of the basin containing it or can be represented by the location and energy of the experimentally-known conformation representing it. The spatial distance d(State, Saddle) is measured via the Euclidean distance in PC1-PC2 space between the conformation representing this state, with *State*∈{*On*,*Off*,*T*−,*R*−,*T*∗−} and the saddle separating the On and Off states (the saddle can be obtained via BDR or SDR). Since results are similar, we relate results with the choice of an experimentally-known conformation representing the state and the saddle reported by BDR. Similarly, an energetic distance dE(State, Saddle) can be defined, measuring the height of the barrier (E(Saddle) - E(State)). Since we track 5 known states of H-Ras, each reconstructed landscape is now summarized with 10 quantitative descriptors and can be represented as a vector of 10 descriptors.

Due to the fact that H-Ras and its mutations are central to human biology and disease, this enzyme of many of its diseased variants have been characterized in wet laboratories. For instance, work in [30, 31] reports biochemical parameters (measured in the wet laboratory) of several catalytic activities in which H-Ras participates, such as GTP activation, GAP sensitivity, (MEK, ERK) activation of the RAF-kinase pathway and AKT activation of the PI3K-kinase pathway, GTP/GDP dissociation, GEF activity of SOS1, intrinsic GTP hydrolysis, GAP-regulated hydrolysis, and RAF1-RBD binding affinity. We number them as P0-P9 (P2-P4 for the three kinase pathways). Work in [30, 31] reports these biochemical parameters for 15 variants of H-Ras (the same variants which we study in this paper): the WT, 2 oncogenic variants (G12V and F28L) and 12 non-oncogenic but syndrome-causing variants (K5N, V14I, Q22E, Q22R, P34L, P34R, T58I, G60R, Y71H, K147E, E153V, F156L).

**Fig. 7:**
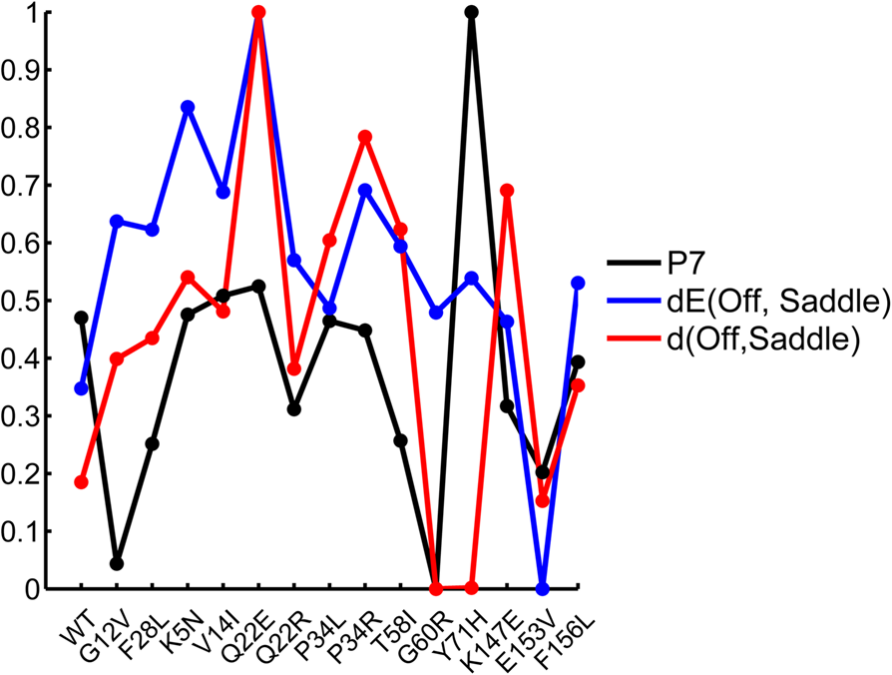
Values of two landscape descriptors and one biochemical parameter are shown across all variants

Leveraging wet-laboratory characterizations of activities of H-Ras variants, we now conduct the following analysis. We compare the extracted landscape descriptors (from the reconstructed landscape of each variant) to the biochemical parameters reported in [30, 31]. Specifically, each of the 10 wet-laboratory biochemical parameters (P0–9) across the variants is compared to each of the 10 landscape descriptors (extracted from reconstructed landscapes as detailed above) across these same variants. Values are normalized as in [*x*− min(*x*)]/[*x*− max(*x*)] to permit a correlation-based analysis. Figure 7 shows two (normalized) landscape descriptors and one biochemical parameter across all 15 variants. Table 1 then lists all landscape descriptor - biochemical parameter comparisons that result in Pearson correlations ≥0.5.

**Table 1:** Measured landscape descriptors and biochemical parameters (reported in [30, 31]) with correlations ≥0.5. T-* indicates the hydrolyzed T-state

State	d(State, Saddle)	dE(State, Saddle)
On	P7(-0.84), P3(0.53)	–
Off	P7(0.83)	P7(0.58), P0(0.54), P8(0.50)
T-	P7(-0.79)	P0(0.62)
R-	P7(-0.85), P3(0.51)	P7(0.61), P0(0.51)
T*-	P7(-0.82)	P7(0.62), P0(0.54), P8(0.53)

Figure 7 and Table 1 allow drawing several insights. As work in [30, 31] reveals, intrinsic hydrolysis is higher in the oncogenic variants over the WT (lower values of P7 correspond to higher intrinsic hydrolysis). The correlation-based analysis provides a possible explanation. Table 1 shows that this occurs due to elevated barriers (positive correlations), movement of the Off state away from the saddle point (positive correlation), and movement of all other states towards saddle point (negative correlations). The correlation-based analysis suggests that equilibrium diffusions from the various states within the On basin to the Off basin directly relate to intrinsic hydrolysis, as this activity is perturbed in pathogenic variants via changes to landscape features.

GAP-catalyzed hydrolysis (P8) is another biological activity of H-Ras on which our analysis provides further insight as to how it is impacted by mutations. Table 1 shows correlations of 0.50 and 0.53 between P8 and Off-to-saddle and hydrolyzed T-to-saddle barrier height variations. These suggest a specific role of the On and hydrolyzed T states in GAP-catalyzed hydrolysis. We note that a prior study relating FoldX energies (FoldX is a protein design method) of specific conformations to biochemical parameters in [30, 31] could only obtain two correlations, 0.65 for intrinsic hydrolysis and 0.43 for GAP-activated hydrolysis [32]. The highest correlations we obtain are −0.85 and 0.58, respectively.

Finally, Table 1 also shows that spatial and energetic distances of states from the On-to-Off saddle point correlate well with parameters that measure GTP activation (P0) and ERK activation of the RAF-kinase pathway (P3). These results suggest that the On and R-states are important for activation of this pathway, and that the increased barrier heights between the GTP-activated states and the saddle point delay activation, thus increasing the amount of unbound GTP in pathogenic H-Ras variants.

## Discussion

As this paper shows, the described methods can be utilized to reveal basins and saddles in an underlying energy landscape. If the focus is on expediently capturing and analyzing saddles, the SDR method is more appropriate, as it employs a rigorous definition of a saddle point. In contrast, the BDR method employs a working definition of saddles and identifies them after extracting the hierarchical organization of basins in the landscape. Doing so is more computationally demanding, and the majority of the computational budget is devoted to identifying basin boundaries via the alpha convex hull. The specific characteristics of basins in a landscape impact performance. For instance, if the landscape is very rugged and contains many basins within basins, the BDR method will spend significant time in extracting this organization. Parameters (such as *δ*_2_, *m*, and *n*_0_) can be leveraged and tuned to control the computational demands at the expense of possibly missing small basins. If the landscape has a shallow hierarchical organization, the computational demands of the BDR method will be low.

Summarizing a protein energy landscape via its basins and saddles, as proposed in this paper, is an interesting venue via which one can pursue relating protein energy landscapes and mutation-driven alterations of landscapes to protein function and dysfunction. Specifically, the analysis presented in this paper allows relating over a dozen mutations of an enzyme key to human biology and health to biological activities via dynamics, validating prior dry- and wet-lab work and revealing novel mechanisms via which mutations percolate to dysfunction. The results presented here are promising and suggest the approach of an exciting stage of landscape-driven enquiry of the relationship between dynamics and function, where one can compute and mine landscapes of protein variants to learn *in-silico* models of how mutations impact function, as well as elucidate the role of specific conformational states and state-to-state interconversions in key biological activities.

## Conclusion

This paper has presented two novel methods that automate the reconstruction and analysis of energy landscapes. In particular, the proposed methods organize the information hidden in evaluated samples and reveal central local structures of a landscape, such as basins and saddles that underly the spatio-temporal dynamics. Our focus on such local landscape structures is due to the role that they play in regulating the equilibrium dynamics of a protein and the insight that they confer on how mutations alter function via altering dynamics.

Finally, it is worth noting that the proposed methods are general and applicable to any set of evaluated samples that populate the state space of a dynamic system. In the strict context of molecular modeling research, the proposed methods can be valuable in analysis of results obtained by tertiary or quaternary structure generation methods, such as template-free protein structure prediction methods, as well as protein-ligand binding and protein-protein docking methods. Analysis of the underlying landscapes populated by decoys representing computed tertiary or quaternary structures can be leveraged to identify thermodynamically-stable and/or semi-stable structural states and possibly advance research in recognition of the native unbound or bound state of a molecule.

## Abbreviations

2D: Two-dimensional
BDR: Basin-driven reconstruction
MC: Monte Carlo
MD: Molecular dynamics
PC: Principal component
PCA: Principal component analysis
PDB: Protein data bank
RGF: Reduced gradient curve following
SDR: Saddle-driven reconstruction
TDA: Topological data analysis
WT: Wildtype
